# Copolymer Considerations on the Displacing Effect for the Effective Functionalization of 3D-Printed Photopolymer Sensors with Carbon Nanotubes

**DOI:** 10.1021/acsomega.5c11529

**Published:** 2026-04-02

**Authors:** Veronika Sevriugina, Petr Lepcio

**Affiliations:** Central European Institute of Technology, 48274 Brno University of Technology, Purkyňova 656/123, Brno 612 00, Czech Republic

## Abstract

This study is part of a research effort motivated by the effective nanofiller functionalization of polymers for 3D printing sensors, enabling the fine-tuning of their functional properties. While the aspects of various conductive fillers, including their morphology, concentration, dispersion, and surface modification, are widely studied, the dispersing medium receives far less attention. Previously, we revealed a significant effect of conjugated electrons on the overall dielectric properties of filled aromatic polymers. This work examines another aspect of the complex interactions in multicomponent nanocomposite resins by investigating homopolymers and copolymers of two nonconjugated acrylic precursors with seemingly similar dielectric properties after curing but different orientational polarizability in the liquid state. Multiwalled carbon nanotubes (MWCNTs) are employed as a model filler. The results linked rheological, dielectric, and photopolymerization properties with performance in a simple capacitance-correlated strain-sensor setup. It revealed nontrivial and unintuitive effects, scaling the functional properties up to an order of magnitude. The best sensors achieved a sensitivity of 57 pF per 1% of strain, linearity of 0.99, and drift below 1.5% after 500 cycles. These were attributed to the adjusted filler dispersion through the displacement effect, which was fueled by the presence of the second monomer acting as a cosolvent. The results guide the path to improved sensor performance and effective nanofiller functionalization while providing critical material considerations for future academic research and industrial development.

## Graphical abstract

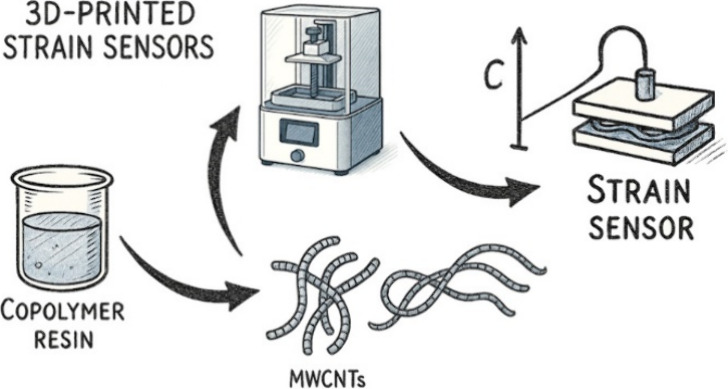

## 1 Introduction

A sensor is a device capable of detecting changes in physicochemical properties, such as temperature, force, mechanical strain, chemical composition, moisture content, or physiological functions, and producing a signal that can be measured and recorded. The working principle of sensors utilizes various effects, such as change of electric potential,^1^ conductance,^2^ resistance,^3^ resonance frequency,^4,5^ electrostatic induction,^6^ piezoelectricity,^7^ or fluorescence.^8^ One of the current trends resonating in sensor research is the on-demand customization fueled by the recent boom of 3D printing.^9^ It allows for the effective adjustment of the design, shape, or even material performance individually to each application.

Three-dimensional printing has recently sprouted as a family of innovative manufacturing methods popular in both research and industry. It can print a wide range of materials, covering metals,^10^ silicates,^11^ polymers,^12^ or composites.^13^ Its application spun from rapid prototyping, customized designs, complex engineering structures, or missing spare parts to high-tech applications, such as energy absorbers, microfluidics, biomedical scaffolds, electronic devices, or sensors.^14–16^ Among them, polymer extrusion and vat photopolymer 3D printers are favored for their relative simplicity, low capital investments, and easy operation. However, most polymers used in 3D printing are electrical insulators, limiting their use as functional materials, including sensors.

This issue could be overcome either by a suitable post-treatment, such as applying a metallic layer on the printout,^17^ or by incorporating a conductive phase in the polymer matrix. The functionalization can target directly the matrix by using conductive polymers, such as poly­(3,4-ethylenedioxythiophene) polystyrenesulfonate (PEDOT:PSS),^1^ yet the number of available materials is limited and, consequently, the range of achievable properties. Research studies more commonly report on the functionalization of polymers with conductive or semiconductive nanoparticles and nanofillers, such as metals,^3^ metal oxides,^18^ graphene,^5^ or carbon nanotubes.^19^ These studies mostly focus on the filler, its morphology, synthesis, chemical modification, concentration limits, dispersion, etc., while considering the polymer matrix mostly just for its mechanical properties. For instance, wearable sensors require flexible and compliant materials,^9^ while strain sensors are usually stiff and robust.

Nevertheless, neglecting the matrix’s properties other than mechanical is very short-sighted.^20^ The strength of polymer–filler physicochemical interaction has been recognized as a critical parameter for achieving the filler’s good dispersion.^21^ That, in turn, influences properties relevant to sensor applications, including the mechanical properties due to the nanoreinforcement effect,^22,23^ as well as the functional electric and dielectric characteristics.^20,24^ For instance, our recent study has revealed that an aromatic high-permittivity polymer with conjugated π electrons, facilitating good electronic interactions with conjugated electrons in carbonaceous fillers, such as carbon nanotubes, was able to detach the electric and rheological percolation of a nanocomposite 3D printing material. As a result, it shifted the conductivity saturation to far lower concentrations.^19^

This study further pursues the motivation for the effective use of functionalized nanocomposites for 3D printing and the ability to fine-tune their dielectric properties. It adds new bits of information to the complex puzzle of interactions in multicomponent nanocomposite resins by comparing homopolymer and copolymer photopolymer matrices while utilizing a fixed concentration of multiwalled carbon nanotubes (MWCNTs) as a model filler. We selected two nonconjugated low-viscosity monomers with good printability as homopolymers, yielding seemingly similar dielectric properties when cured. However, their orientational polarizability in the uncured state differs significantly. Both monomers facilitated a relatively good dispersion in the liquid state, which was then fixed by photopolymerization upon 3D printing. Rheological, dielectric, and photopolymerization properties were correlated to strain-sensor performance based on a simple capacitance measurement under cyclic mechanical loading at two different dielectric frequencies. The results revealed a strong displacing effect, i.e., a preferential interaction with one of the monomers, determining the functional properties, such as permittivity, conductivity, or dielectric loss, by up to an order of magnitude. That, in turn, translated into the sensor performance in a simple capacitance-correlated strain testing setup.

## 2 Materials and Methods

### 2.1 Materials

Two low-viscosity difunctional acrylate monomers, tricyclodecanedimethanol diacrylate (SR833S) and propoxylated 2-neopentyl glycol diacrylate (SR9003), were supplied by ARKEMA Sartomer, NL. Phenylbis­(2,4,6-trimethylbenzoyl)­phosphine oxide (BAPO) from RAHN, Switzerland, was selected as a photoinitiator. Nanocyl, S.A (Belgium), provided multiwalled CNTs (MWCNTs) NC7000, manufactured by a catalytic chemical vapor deposition process and purified to an average carbon purity of 90%. The average diameter of a nanotube is 5 nm, and its average length is 1.5 μm. A more detailed characterization can be found elsewhere.^19^ All materials were used as received without modification or purification.

### 2.2 Sample Preparation

Resins were prepared by magnetic stirring at 60 °C for 1 h to mix the monomers according to Table 1 and dissolve the photoinitiator (3% w/w). The resins were handled under yellow-light conditions to prevent premature curing in ambient light.

**Table 1:** Overview of Sample Compositions

	amount, % w/w		
	monomer mixture		filler
sample code	SR833S	SR9003	MWCNTs
SR8	100		0.21
SR8-5SR9	95	5	0.21
SR8-10SR9	90	10	0.21
SR8-20SR9	80	20	0.21
SR8-30SR9	70	30	0.21
SR9	100		0.22

The MWCNT concentration of 0.25 vol % was selected as a model filler. The filler’s weight concentration was recalculated according to mixtures’ densities and is provided in Table 1. According to our previous research, it represents the optimum nanofiller loading that could be processed easily by simple vat 3D printers due to gelation and light absorption at higher concentrations.^19^ MWCNTs were added to the neat matrix, first mixed by a magnetic stirrer (IKA RCT Basic) for 10 min and then dispersed by an ultrasonic probe homogenizer (Bandelin Sonopuls HD 3200) to ensure their uniform distribution. The average energy input for prepared nanocomposites was 95.1 ± 10.6 J/ml.

The formulations were 3D printed with an Original Prusa SL1S vat photopolymerization 3D printer equipped with an LED light source (VPP-LED). The printer’s irradiance was 2.07 mW·cm^–2^ with the maximum emission intensity at 405 nm. The printing instructions were generated in the OEM software Prusa Slicer (v. 2.9.0). Testing specimens of flat disks with diameters of (25 × 1) mm^2^ were sliced with a layer thickness of 50 μm. The exposure time was set to 20/5 s for the first/other layers in neat resins and 45/15 s in nanocomposites. The 3D-printed samples were analyzed without additional curing.

### 2.3 Rheology

Rheological properties were investigated with an advanced rotational rheometer (DHR-2, TA Instruments, USA) at 30 °C isothermal conditions using a 40 mm parallel-plate geometry and a gap of 1 mm. Oscillatory frequency and strain amplitude tests were performed. According to the empirical Cox–Merz rule, the steady-state viscosity’s shear-rate dependence is equivalent to the complex viscosity’s angular frequency dependence. The power law index was evaluated from the complex viscosity obtained in a frequency sweep test ranging from 0.1 to 20 Hz at a strain of 0.1%. The power law index *n* signals the viscosity depression in the shear-thinning power law region (eq 1):

$$\eta \left(\gamma^{\prime }\right)=k\gamma^{\prime n-1}$$ 1

The variables η, γ′, and *k* represent viscosity, shear rate, and consistency, respectively.^25^ On top of that, the 1 Hz linear viscoelastic region (LVR) complex viscosity η_1 Hz_ was determined from the linear part of the strain amplitude sweep test performed at the strain amplitude ranging between 0.1% and 50% and the reference frequency of 1 Hz.

### 2.4 Scanning Electron Microscopy

The 3D-printed samples were cryofractured in liquid nitrogen to prevent plastic deformation and coated with 10–12 nm of sputtered gold using ACE 600 (Leica microsystems, Germany). The fracture faces were then observed with a Mira 3 XMU scanning electron microscope (Tescan, Czechia) at 30 kV using secondary electron detectors.

### 2.5 Dielectric Thermal Analysis

Dielectric properties were investigated with an ARES-G2 rheometer (TA Instruments, USA) equipped with an OEM dielectric accessory. It consists of 25 mm parallel plates with built-in electrodes and ceramic insulation using an Agilent E4980A precision LCR meter for field control and measurement. Dielectric permittivity data were collected from uncured liquid resins and 3D-printed discs at a 1 mm gap. The oscillating voltage signal of 1 V was used at frequencies from 20 Hz to 2 MHz under isothermal conditions at 30 °C. In addition, an axial force of 1 N was applied to the printed disks to obtain good contact between the solid specimens and the geometries.

The determined frequency-dependent complex dielectric permittivity ε* consists of the real part represented by the relative dielectric constant ε′ and the imaginary part known as the dielectric loss ε″ (eq 2):^26^

$$\varepsilon^{*}=\varepsilon^{\prime }-{i\varepsilon }^{\prime \prime }$$ 2

where *i* is the imaginary number. The dielectric loss factor tan δ was obtained as a function of frequency given by eq 3:^26^

$$\tan\;\delta =\frac{\varepsilon^{\prime \prime }}{\varepsilon^{\prime }}$$ 3

### 2.6 Jacobs Working Curves

Jacobs working curves assess the photocurability of the material through a Beer–Lambert relationship, derived from a logarithmic linear plot of cure depth (*C*_d_) and the curing energy (*E*_0_), given by the printer’s irradiation intensity (*I*_0_) and curing time (*t*). The critical energy (*E*_c_) required to initiate the formation of a solid layer is calculated as the intersection of the Jacobs working curve with the *x* axis, while the slope defines the penetration depth (*D*_p_) according to eq 4:^27,28^

$$C_{d}=D_{p}\cdot \ln\left[\frac{E_{0}}{E_{c}}\right]=D_{p}\cdot \ln\left[\frac{I_{0}t}{E_{c}}\right]$$ 4

Jacobs working curves were obtained by illuminating (10 × 10) mm^2^ squares in the absence of the printing platform for varying exposure times and measuring the cure depth with a micrometre.

### 2.7 Dielectric-Strain Response

The dielectric-strain response was measured with the RSA-G2 instrument (TA Instruments, USA) equipped with 25 mm parallel-plate electrodes connected to the Agilent E4980A precision LCR meter. The 1 mm thick specimens were tempered at 30 °C to avoid the necessity of cooling with a nitrogen medium, which would affect the measured permittivity. The sample was loaded and unloaded in compression in 10 cycles at a linear strain rate of 0.001 s^–1^ (≈0.1%·s^–1^) and amplitude of 120 s. The maximum strain of 6% was achieved at the middle of the cycle (at 60 s). We note that the testing strain mode was bending rather than genuine compression due to the inherent curvature of the printouts and the detected modulus of only tens of kPa.

The dielectric properties were measured simultaneously during the loading by applying an oscillatory field with a voltage of 1 V and a frequency of 1 kHz for the first five cycles immediately followed by another five cycles at 1 MHz. The sensor’s sensitivity was established from the change of capacitance per unit strain from each half-cycle. The linearity was assessed by the *R* ^2^ parameter obtained from a linear fit of each capacitance-stress half cycle. Finally, the repeatability was calculated as the average signal deviation from the first cycle of each measurement.

Long-term stability was determined as the drift in the sensitivity at 1 kHz, i.e., the slope of capacitance change with strain, between 4% and 6% of deformation. Three measuring cycles were followed by 97 nonmeasuring harmonic fatigue cycles until a total of 503 cycles elapsed. The period of each cycle was 120 s, while the voltage of 1 V was constantly applied during the whole measurement.

## 3 Results and Discussion

A typical layer thickness in vat photopolymerization 3D printing is around several tens of microns. Hence, the printing stage incrementally moves to make space for a new layer, while the resin must adapt to platform movements by flowing, making viscosity a prominent parameter in this process. However, viscosity is not a static property but often scales with the applied shear rate. This could be expressed by a power law (eq 1) where the value of 1 means a Newtonian shear-rate independent behavior, while higher and lower values denote non-Newtonian shear thickening and shear thinning response, respectively. The viscosity deviations express the change in internal friction, strongly correlating with the inner attractive and repulsive forces of the investigated liquid. Hence, the viscosity and power law index are powerful tools for studying the internal processes at the molecular scale.^25,29^

SR833S and SR9003 were selected as representative nonconjugated difunctional low-molecular-weight low-viscosity monomers with good printability. SR833S (tricyclodecanedimethanol diacrylate) contains a nonaromatic cyclo-alkyl structure, while SR9003 (propoxylated 2-neopentyl glycol diacrylate) is noncyclic and slightly more polar. The SR8 and SR9 resins (with a BAPO photoinitiator, Table 1) had zero shear viscosities of 113 and 15 mPa·s, respectively (Figure 1a). SR8 was weakly shear thinning, while SR9 was shear thickening, as documented by the power law index (Figure 1b). A systematic viscosity decrease was observed for diluting the SR8 resin with the SR9003 monomer in the order SR8 > SR8-5SR9 > SR8-10SR9 > SR8-20SR9 > SR8-30SR9 > SR9 (Figure 1a). Nevertheless, the measured viscosities of the copolymer resins (SR8-5SR9, SR8-10SR9, SR8-20SR9, and SR8-30SR9) were lower than the mathematical average of SR8 and SR9 resins, indicating a limited affinity between the two monomers^25^ (Figure 1a).

**Figure 1:**
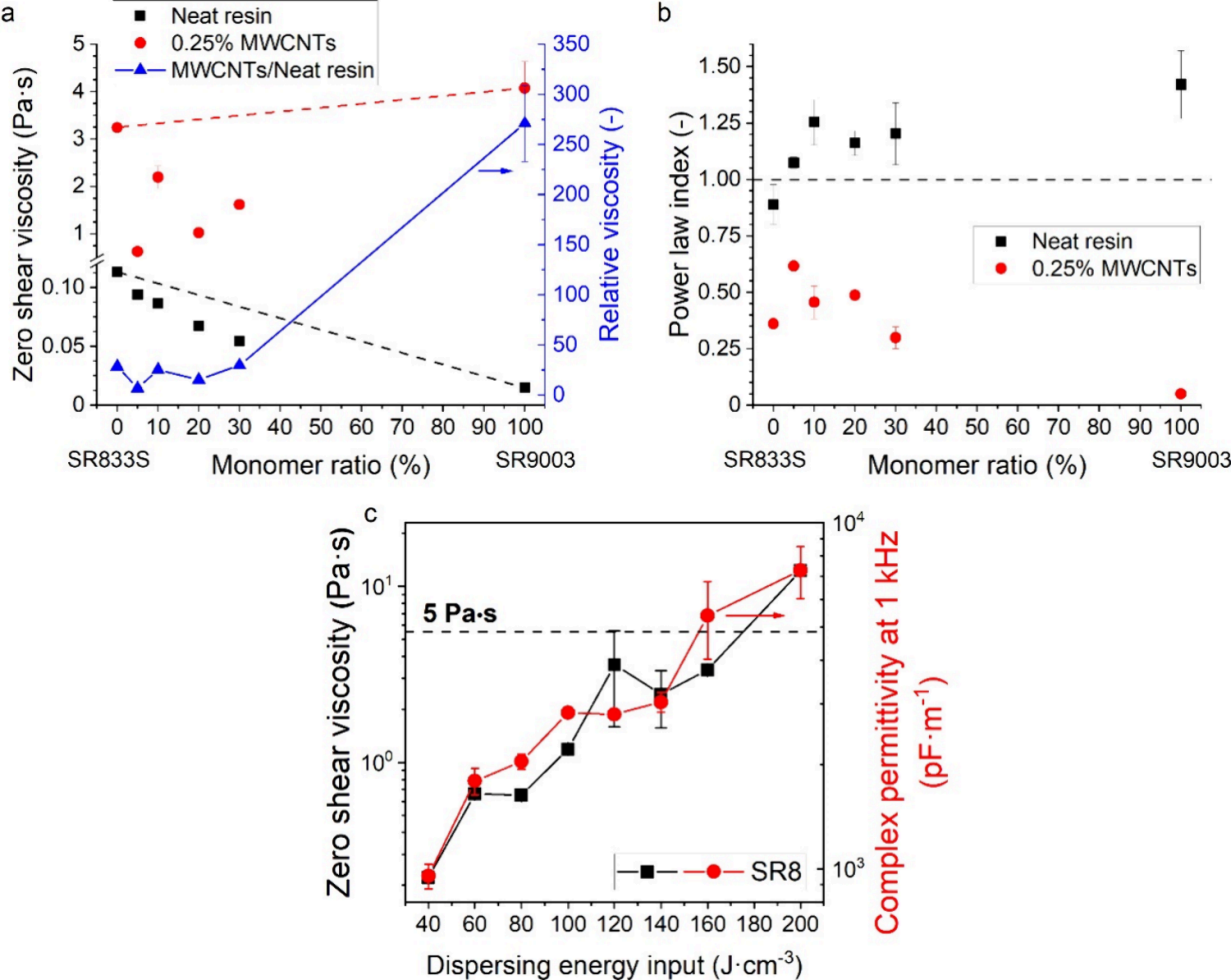
Zero shear viscosity (a) and power law indices (b) as a function of the monomer ratio. (c) Zero shear viscosity and complex permittivity at 1 kHz of the SR8 resin with 0.25% MWCNTs as a function of energy input applied by the ultrasonic probe to disperse MWCNTs.

The situation was perplexing after adding multiwall carbon nanotubes (MWCNTs) as a representative functional nanofiller. Nanomaterial additives typically increase the viscosity due to chain adsorption-mediated nanoreinforcement effect, often to a point where the resin can no longer be printed by standard vat printers or even flow.^19,30^ The prints often start to be defective when the viscosity exceeds several pascal seconds (Pa·s), but the exact value depends on the platform movements of the specific printer and the size and shape of the gap between the illumination screen and the printing platform with the previously printed layers. The performance can be partially adjusted by the shape, orientation, and placement of the printed objects and the layer thickness, but too large viscosity inevitably leads to bubbles entrapped in the resin, shape distortion due to thermomechanical relaxation, or delamination/detachment of the printed object due to suction forces when the platform moves while preparing to print the next layer.

Moreover, nanofillers often shorten the photocurable resin’s penetration depth due to light absorption, as will be discussed later. Since this work focuses on the interactions within the polymer matrix, the filler concentration was kept at a constant value of 0.25% vol/vol^19^ to ensure good printability in various resin formulations. A detailed study of the filler concentration dependence was reported in our previous work.^19^ In short, the concentration of 0.25% vol/vol was above the percolation limit for MWCNTs dispersed by the ultrasonic probe. The complex permittivity was significantly increased, especially for the high-permittivity matrix with delocalized π electrons, while a weaker enhancement was observed in the matrix with lower permittivity.

MWCNTs, like all nanomaterials in a dry state, were aggregated and required dispersing by a strong force input, such as cavitation induced by high-energy ultrasonic probes.^30^ The zero-shear viscosity and complex permittivity, i.e., parameters sensitive to the dispersion quality, scaled systematically with the input energy per unit volume (Figure 1c). Long ultrasonication times promote the dispersion of MWCNTs to a point where the formulations start forming a gel, deteriorating their printability. Therefore, the average energy input was set to 95.08 ± 10.56 J/ml to ensure good repeatability of the results.

The zero-shear viscosity and power law index indirectly indicate the filler’s dispersion quality^19^ and the balance of internal forces,^25^ which are two closely related parameters.^21^ All filled resins showed a power law index less than 1, indicating the presence of a relatively weak load-bearing network formed by the MWCNTs that can be disrupted under shear.^31^ Interestingly, the highest viscosity of 4.1 Pa·s was observed for the filled SR9 resin (Figure 1a), which was also the least viscous composition among the unfilled resins (113 mPa·s). This sample also had the lowest power law index (0.05, Figure 1b). The second most viscous resin was SR8 with MWCNTs (3.2 Pa·s, Figure 1a) but with a slightly higher power law index (0.36, Figure 1b). All mixed-monomer formulations were less viscous than the single-monomer ones when filled with MWCNTs (Figure 1a), with power law indices between 0.30 and 0.62 (Figure 1b).

Since a high increase in viscosity and low value of the power law index were previously correlated with good filler dispersion,^19^ these results suggest that the best dispersion is achieved in single-monomer formulations (SR9 and SR8), as confirmed by the SEM images (Figure 2). Nanofillers in two-component dispersants were previously documented to organize in complex structures^30^ due to the second solvent acting as a displacer, modifying the effective force balance on the filler’s surface,^32^ which controls the dispersion state. That is particularly evident in the SR8-5SR9 sample, which formed thick bundles (Figure 2b), leading to low viscosity and high power law index (Figure 1).

**Figure 2:**
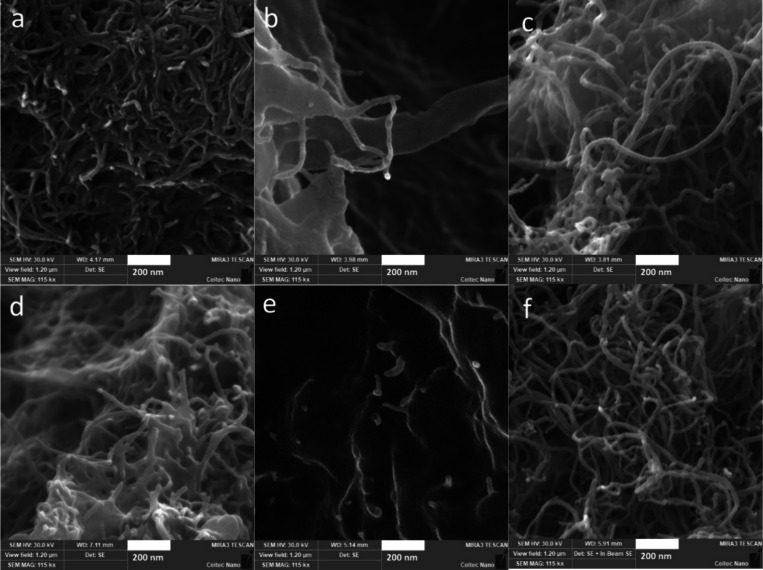
SEM images of MWCNTs in (a) SR8, (b) SR8-5SR9, (c) SR8-10SR9, (d) SR8-20SR9, (e) SR8-30SR9, and (f) SR9 samples. The scale bars represent 200 nm.

Dielectric properties represent important parameters of the functionalized nanocomposite resins governing their performance in microelectronics, sensors, energy storage, batteries, or capacitors. Moreover, they offer insight into charge carriers, ionic conductivity, and ion mobility, which refer to the material’s inner structure and the structural changes during the photopolymerization.^24,33^ The individual frequency-dependent contributions consist of interfacial (above approximately 10^–6^ Hz), ionic (≳10^–3^ Hz), dipole (≳10^3^ Hz), vibrational (≳10^12^ Hz), and electronic polarization (≳10^15^ Hz).^34,35^ The polarizability is quantified by dielectric permittivity ε at different frequencies (Figure S1).

When the sample is subjected to an external electric field oscillating at a frequency of 1 MHz ≈ 10^6^ Hz, the interfacial, ionic, and dipole polarizations are too slow to contribute to the overall polarizability. Thus, the complex permittivity of liquid resins at 1 MHz (ε_1 MHz_) represents a measure of electronic and vibrational polarization (Figure 3a). The electronic polarizability occurs in all types of dielectric materials and scales with the number of polarizable electrons.^35^ The difference between the polarizability at 1 MHz ≈ 10^6^ Hz and 1 kHz ≈ 10^3^ Hz could be attributed to dipolar polarization, also known as orientational polarization, which is the primary form of polarization in dipolar dielectrics.^35^ Furthermore, interfacial polarization can be considered as a combination of conduction and dipolar polarization mechanisms in materials with a conductive phase dispersed in an insulating matrix.^36^ Intermolecular forces trap charged species at the interface, which can absorb electromagnetic waves by a mechanism similar to that of dipolar polarization. The restrictive forces induce a phase lag under oscillating field, generating random motion of trapped ions and dissipating significant energy.^36^ The interfacial polarization significantly contributes to the dielectric properties of polymer composites filled with high permittivity fillers, such as titanium dioxide or carbon nanotubes.^20^

**Figure 3:**
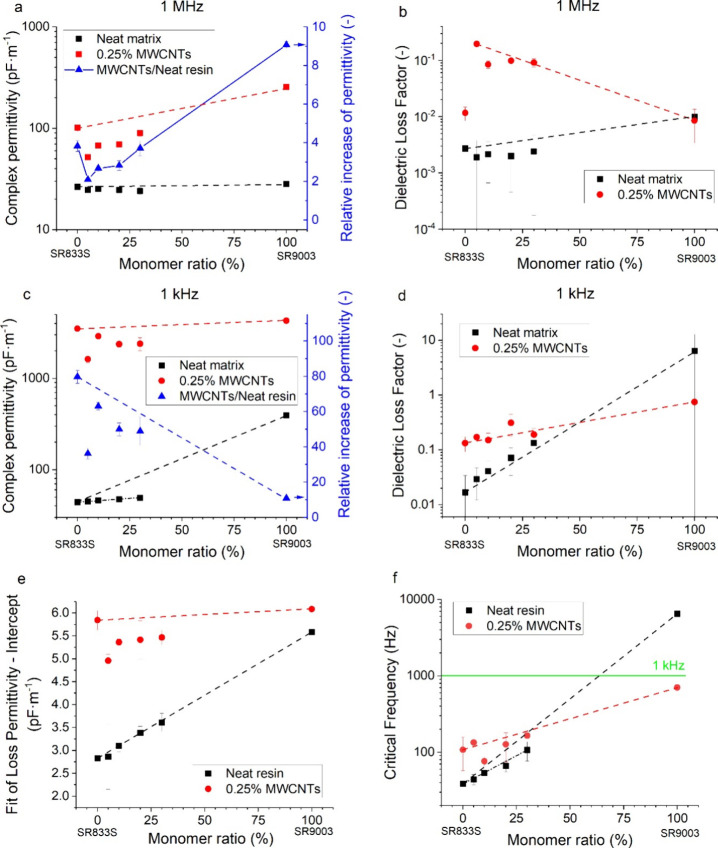
(a, c) Complex permittivity and its relative increase upon addition of MWCNTs, (b, d) dielectric loss factor at (a, b) 1 kHz and (c, d) 1 MHz, (e) intercept fitting parameter of loss permittivity, and (f) critical frequency of complex permittivity trend change for the uncured nanocomposite resins. The dashed lines indicate the mathematical average between the SR8 and SR9 samples. The dashed–dotted lines indicate the mathematical average between SR8 and SR8-30SR9 samples.

The ε_1 MHz_ of neat resins varied between 43.7 and 61.4 pF·m^–1^ (Figure 3a). The SR9 was slightly more polarizable (59.1 pF·m^–1^) than SR8 (43.7 pF·m^–1^), correlating with six oxygen atoms in the propoxylated 2-neopentyl glycol diacrylate (SR9003) compared to only four oxygen atoms in tricyclodecanedimethanol diacrylate (SR833S). The mixed monomer resins scaled linearly with the monomer concentrations (44.6, 45.6, 47.2, and 48.4 pF·m^–1^, respectively) with an *R* ^2^ of 0.999, indicating no electronic interactions between the monomers (Figure 3a). Accordingly, low loss factors (≤0.07) were recorded for all resins (Figure 3b) due to very low values of loss permittivity (ε″).

SR9 showed a significant orientational polarizability at 1 kHz (ε_1 kHz_ = 394.3 pF·m^–1^, ε_1 kHz_/ε_1 MHz_ = 6.68; Figure 3c) associated with a very high loss factor (6.44, Figure 3d), while no difference was observed for SR8 (ε_1 kHz_ = 44.0 pF·m^–1^, ε_1 kHz_/ε_1 MHz_ = 1.01). These results indicate free dipole rotation in the SR9 under an electric field.^35^ The good polarizability is contributed by the SR9’s aliphatic noncyclic structure and lower viscosity (Figure 1a), allowing the good dipole rotation. It also likely corresponds with the rheologic-associative behavior observed for this monomer due to enhanced dipole–dipole interactions when the molecules get oriented in the shear field (Figure 1b). On the contrary, stiffer monomers and a viscous environment can restrict the dipole orientation and reduce the polarizability. Despite the rheologic-associative behavior being preserved in the copolymer resins, the orientational polarization at 1 kHz effectively diminished upon addition of the second monomer (ε_1 kHz_/ε_1 MHz_ ≈ 1.01). The permittivities of these samples are still dominated by the storage permittivities (Figure S1) as indicated by the low loss factors (Figure 3d).

The frequency dependence (Figure S1) revealed a practically constant storage permittivity (ε′), while the loss permittivity (ε″) showed a nonlinear behavior between ≈10^5^and 2·10^6^ Hz but increased steadily in a power law dependence between 20 and ≈10^5^ Hz (Figure S1). The loss permittivity data, appearing as a straight line in the log–log plot, were fitted with a linear regression (log *y* = *a*·log *x* + *b*). The slope *a* retained a value near −1, but the intercept *b* increased systematically from 2.83 for S8 to 5.09 for S9 (Figure 3e), indicating a higher number of orientable dipoles with increasing SR9003 content. Consequently, the critical frequency below which the complex permittivity becomes dominated by the loss contribution shifts from 39 Hz for SR8 to nearly 6.5 kHz for SR9 (Figure 3f). This factor is responsible for the seemingly large deviation of the SR9’s performance at 1 kHz from the remaining samples (Figure 3c,d)

MWCNTs increased the permittivity unevenly, introducing several nonlinearities (Figure S1). The smallest enhancements of ε_1 MHz_ (Figure 3a) were observed for SR8-5SR9 (28.1×) and SR8-20SR9 (36.7×), while the greatest changes were found in SR8 (61.7×), SR8-10SR9 (49.7×), and SR9 (43.6×). However, the increase in permittivity was associated with higher dielectric losses (Figure 3b), which are typical^20^ for leakage currents and confirm the presence of conductive paths between the fillers in contact.^33^ The only exception was SR9, where the MWCNTs slightly decreased the dielectric loss factor from 0.010 to 0.009 due to the combination of good dispersion and electronic interactions between the monomer and the nanofiller (Figure 3b). The second lowest dielectric loss factor among the filled resins was detected in SR8 (0.012), while it was significantly higher (0.084–0.099) in the copolymer resins, correlating well with the viscosity results (Figure 1), SEM images (Figure 2), and the conclusions regarding the filler dispersion.

The ε_1 kHz_ values of the SR8 (3506 pF·m^–1^) and SR9 (4288 pF·m^–1^) samples with MWCNTs are much more alike than those of the neat resins (Figure 3c), suggesting the dominant role of the MWCNT network over the relatively minor contribution of the monomer’s dielectric properties. Such behavior is in line with ferroelectric (i.e., spontaneously electrically polarized) or conductive domains due to space charge accumulation at the interface and correlates with previous studies claiming the interfacial polarization as the dominant polarization mechanism of carbon nanotubes-filled polymer composites at approximately 1 kHz and below.^20^ Interestingly, MWCNTs significantly reduced the loss factor (0.752) of the SR9 (6.442) by suppressing the orientational polarization due to the monomer–filler interaction. The ε_1 kHz_ of the copolymer resins was below the mathematical average of the SR8 and SR9 samples (Figure 3c), further supporting the conclusion of their worsened dispersion compared to the single-monomer formulations (SR8 and SR9). Interestingly, the dielectric loss factor scaled systematically with the addition of the more polar SR9 monomer (Figure 3d). These findings might be interesting for tuning the performance of sensors relying on high permittivity and high dielectric loss factor.^4^

Nevertheless, the sensors are usually used in the solid state, bringing attention to the curing properties of the nanotube-functionalized photopolymers. The depth of the cured polymer grows with the exponent of the exposure energy (Figure 4a), which is defined by the printer’s irradiance and exposure time (eq 4). The SR8 resin has a lower critical energy (4.36 mJ·cm^–2^) and higher penetration depth (176 μm) than SR9 (7.11 mJ·cm^–2^ and 153 μm, respectively) (Figure 4b,c). The values were derived from the Jacobs working curves according to eq 4. Consequently, SR9 requires longer printing times than SR8 (Figure 4d). The trend in copolymer resins is blurred by experimental error but suggests a dominant contribution of SR9 to the penetration depths (Figure 4b), while the critical energy was close to that of SR8 (Figure 4c).

**Figure 4:**
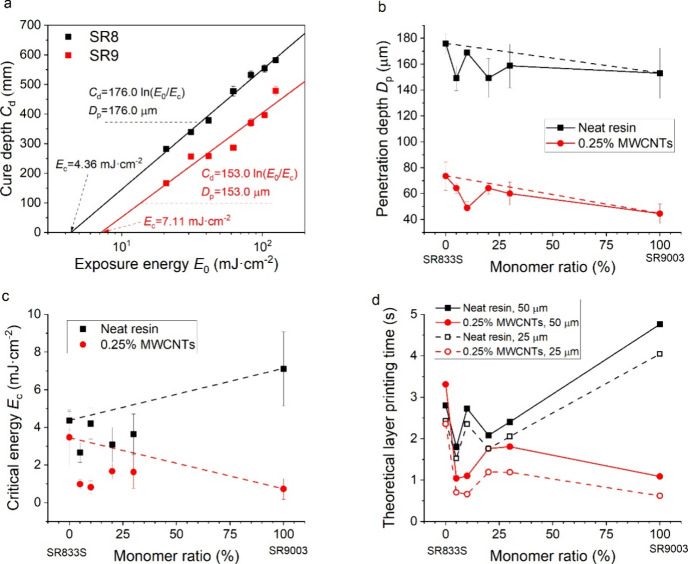
(a) Example of Jacobs working curves for neat photocurable resins SR8 and SR9, (b) penetration depth, (c) critical energy, and (d) layer printing times of neat and filled photopolymer resins.

MWCNTs decreased both the penetration depth (Figure 4b) and the critical energy (Figure 4b), consistent with our previous reports on nanocomposite photopolymers.^19,24,37,38^ Since these two effects contribute antagonistically to printing speed, we plotted estimated times to cure typical printing layers with heights of 25 and 50 μm (Figure 4d). MWCNTs mostly decreased the theoretical printing time, except for the SR8, where only a small reduction of the critical energy was observed. Interestingly, the lowest critical energy (0.73 mJ·cm^–2^) and penetration depth (44.6 μm) were observed in SR9-MWCNTs, inverting the trend observed in neat resins and aligning with the expected good dispersion.

The dielectric properties of both unfilled homopolymers in the solid state, i.e., after curing, are seemingly similar. Complex permittivity (Figure 5a,b) drops significantly compared with the uncured liquid state (Figure 3a,c). Incorporating the monomer molecules into a covalently bonded polymer network confines their free movement, while dipole–dipole interactions suppress polarizability.^35^ The ε_1 MHz_ reduced upon curing from 43.7 to 26.5 pF·m^–1^ (SR8) and from 59.1 to 28.3 pF·m^–1^ (SR9), nearly erasing the contribution of additional oxygens in the propoxylated 2-neopentyl glycol diacrylate (SR9003). A larger change is observed for ε_1 kHz_, where curing suppressed the orientational polarization of SR9 (394.4 pF·m^–1^), yielding only a slightly higher value (32.7 pF·m^–1^) than that of SR8 (28.4 pF·m^–1^) after curing (Figure 5b). The dielectric loss remains low at both 1 MHz (0.02–0.05, Figure 5c) and 1 kHz (0.01–0.03, Figure 5d), while the conductivity belongs to the semiconductive and insulating range at 1 MHz (Figure 5e) and 1 kHz (Figure 5f), respectively. However, in terms of mechanisms, the materials should be considered as insulators.

**Figure 5:**
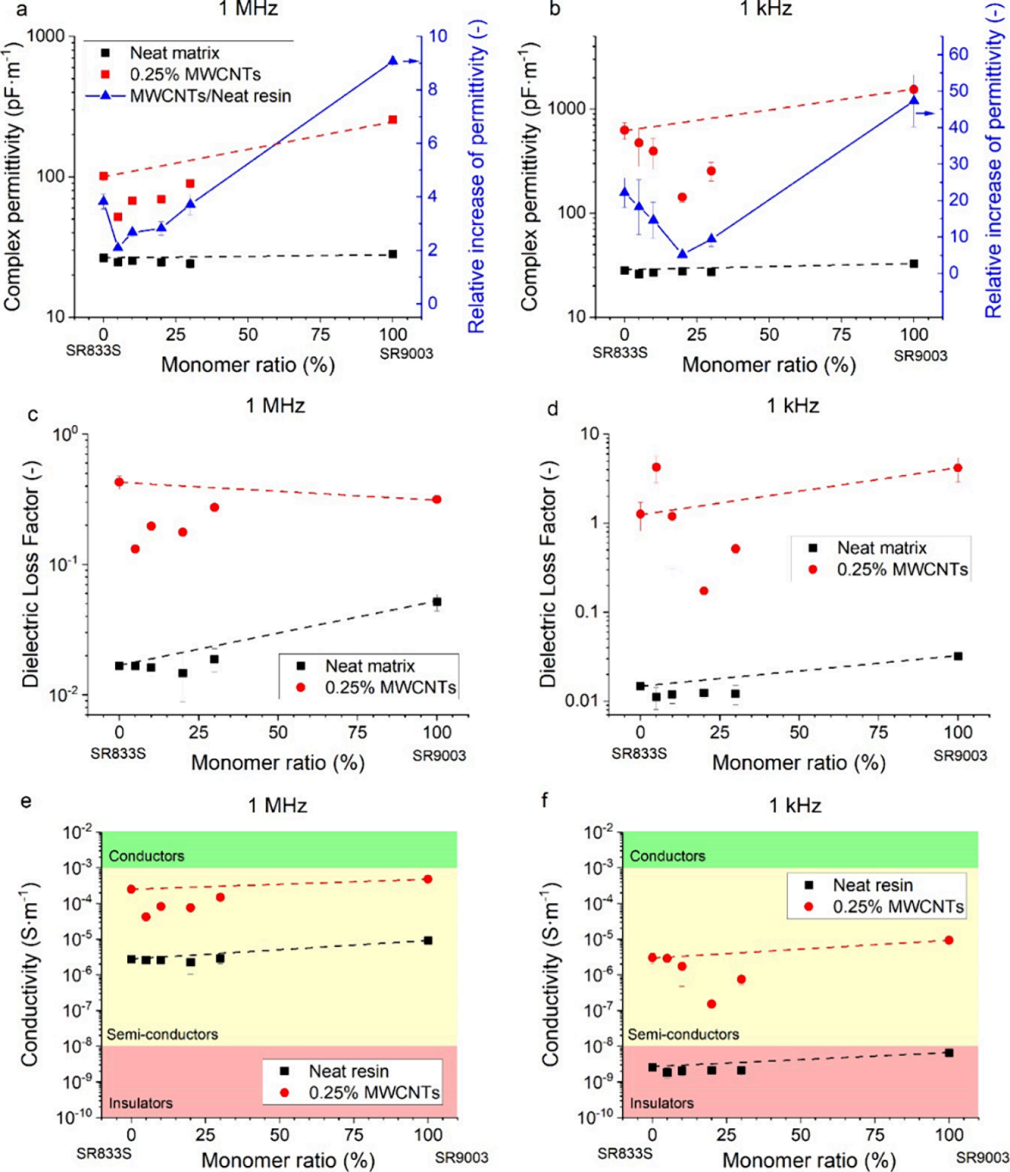
(a, b) Complex permittivity, (c, d) dielectric loss factor, and (e, f) conductivity at (a, c, e) 1 MHz and (b, d, f) 1 kHz for the 3D-printed samples (solid state).

Despite the neat formulations having very similar dielectric behavior when cured, significant differences were observed after functionalization with MWCNTs. Complex permittivity, dielectric loss factor, and conductivity were all significantly promoted at both 1 kHz and 1 MHz (Figure 5a–f). However, the change was nontrivial. The ε_1 MHz_ and ε_1 kHz_ of SR9 increased 9.1 and 47.3 times, respectively, while they rose only 3.8 and 22.2 times for the SR8 resin, respectively (Figure 5a,b). The difference in the electric conductivity of the matrix and filler entraps free charge carriers that accumulate at the interface.^33^ The more pronounced enhancement observed at 1 kHz suggests that the interfacial charge can be polarized (Figure 5a,b). It is worth noting that the monomer–filler interface already exists in the liquid state, where the monomer orientability and polarizability are still high, as documented in Figure 3. Yet, it is unclear what changes to this interfacial region are imposed during the polymerization.

Apparently, the tested homopolymer dielectrics were more effectively functionalized by the MWCNTs than the copolymer ones, while the more polar and flexible propoxylated 2-neopentyl glycol diacrylate (SR9) was superior to the tricyclodecanedimethanol diacrylate monomer (SR8). The copolymer resins showed lesser enhancement at both frequencies (Figure 5a,b), and the permittivity dependence on the monomer concentration was nonmonotonic, with the lowest value achieved for SR8-5SR9 at 1 MHz (2.1 times) and SR8-20SR9 at 1 kHz (5.2 times). These samples were also associated with the worst filler dispersion. In contrast, many commercial 3D printing resins are mixtures of several different monomers, where the interplay of forces may be too complex to predict easily.

These effects were attributed to the polymer–filler interfacial interactions and, consequently, the quality of the filler dispersion. The high dielectric loss factors observed in SR8 and SR9 samples with MWCNTs (Figure 5c,d) further support these conclusions since an interconnected conductive network yields higher losses due to current leakage^33^ as indicated also by the conductivity data (Figure 5e,f). A high dielectric loss is usually considered disadvantageous for most dielectric purposes.^34^ However, it represents a preferable option for specific dielectric sensor applications, especially in combination with high permittivity.^4^ On the other hand, dielectric loss can be reduced more effectively by suppressing filler contact with a surface modification.^39^ All of these aspects must be considered since the proper choice of material composition can improve the final permittivity by up to an order of magnitude. Alternatively, the monomer composition can be used to fine-tune the dielectric properties since the properties scale less dramatically than with the filler concentration.

The 3D printed discs functionalized with MWCNTs were tested for sensing strain with a simple compression setup. We note that the testing strain mode was more akin to bending than genuine compression due to the inherent curvature of the printouts and the detected modulus of only tens of kPa. Nevertheless, this primitive testing setup, loading five cycles while simultaneously measuring dielectric properties at 1 kHz followed by another five cycles at 1 MHz, allowed us to compare the sensing performance of different materials. An example of such a measurement is shown in Figure 6a.

**Figure 6:**
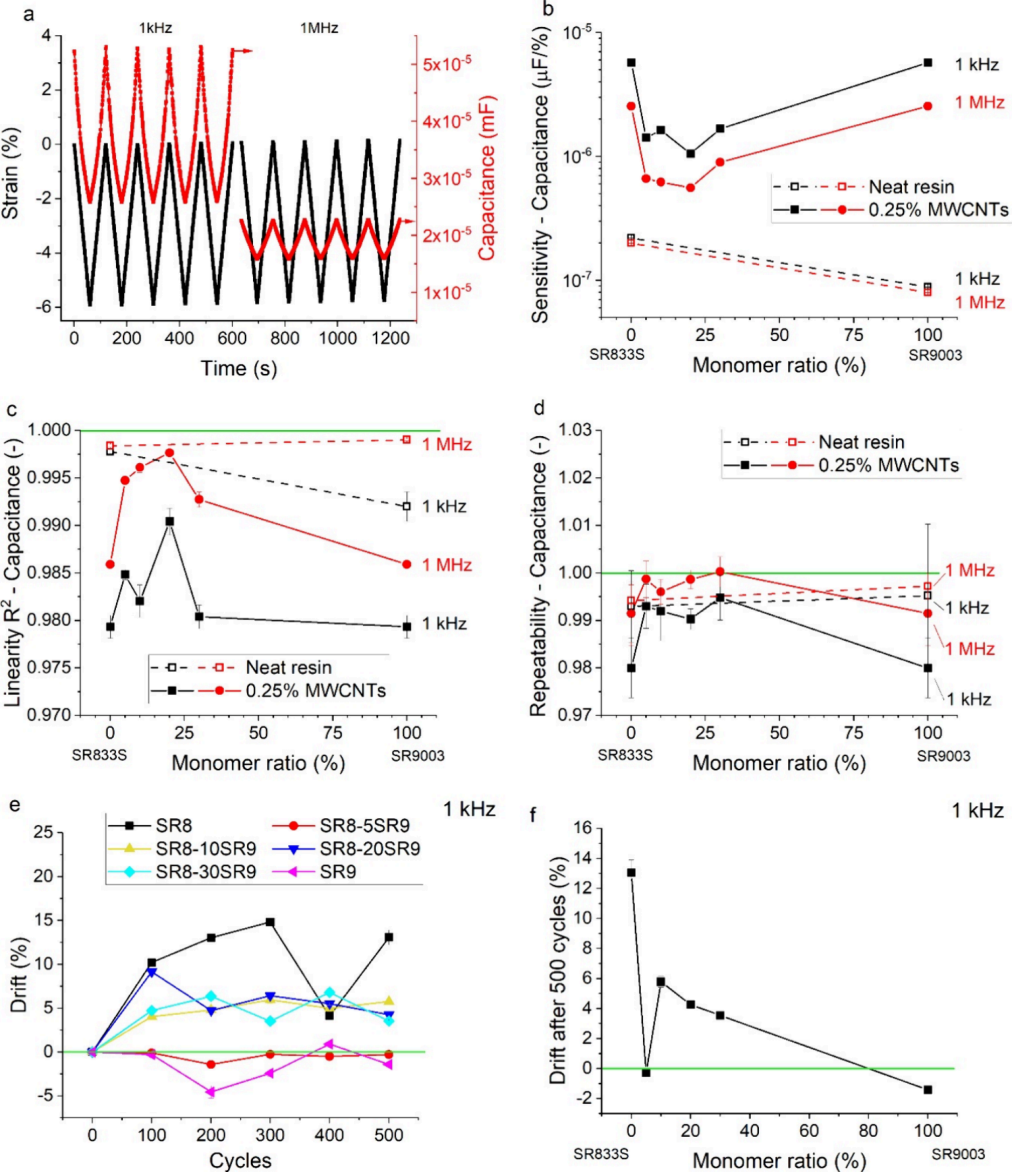
(a) Example of a cycled linear mechanical strain coupled with dielectric measurement at 1 kHz (first five cycles) and 1 MHz (last five cycles), (b) sensitivity, (c) linearity, and (d) repeatability of the capacitance signal as a function of strain. (e) Drift in the capacitance sensitivity at 1 kHz as a function of elapsed cycles and (f) final drift after 500 cycles as a function of monomer ratio. The green line in panels c, d and f represents ideally linear and repeatable behavior, respectively.

MWCNT functionalization increased the sensitivity of the capacitance response by 1–2 orders of magnitude, reaching values of up to 57 and 25 pF/% at 1 kHz and 1 MHz, respectively (Figure 6b). The sensitivity was higher at 1 kHz due to the interfacial polarization, as discussed above. Interestingly, the higher permittivity of the SR9-MWCNT sample (Figure 5a,b) was not translated into higher sensitivity, which was comparable to that of SR8-MWCNTs (Figure 6b). Copolymer formulations reduced the sensitivity 2.5–4.5 times at 1 MHz and 3.4–5.4 times at 1 kHz (Figure 6b), suggesting that good MWCNT dispersion is favorable for enhancing sensitivity. On the other hand, the SR8 and SR9 sensors yielded the worst linearity (≈0.98 at 1 kHz, ≈0.985 at 1 MHz; Figure 6c) and repeatability (≈0.98 at 1 kHz, ≈0.99 at 1 MHz; Figure 6d) among the tested samples. This behavior can possibly be attributed to the leakage currents and high dielectric loss factors of the homopolymer samples (Figure 5c,d), favoring the separated fillers for more ideal dielectric performance, as suggested in the literature.^39^ Moreover, the matrix composition affected the long-term stability represented by the drift in the sensor’s sensitivity after cycling (Figure 6e). While the SR8-MWCNT sample has lost 13.1% of sensitivity after 500 cycles, the SR9-MWCNT sample was 1.4% more sensitive after cycling (Figure 6f). This observation suggests that strong interactions of the MWCNTs with the orientable dipoles of the SR9 monomer help facilitate good filler dispersion in the liquid resin and stabilize it for mechanic cycling in the solid state.

## 4 Conclusions

This research investigates the aspects of internal homopolymer and copolymer interactions in photopolymer 3D printing formulations functionalized with multiwall carbon nanotubes (MWCNTs). It considers several important material aspects for monomer selection, particularly in relation to its functional properties. Foremostly, many important processing and functional parameters, such as viscosity, critical curing dose, penetration depth, permittivity, dielectric loss, or conductivity, are directly related to the filler dispersion, which, in turn, is affected by the input of dispersing energy and internal molecular interactions. A suitable photopolymer composition can enhance the properties by up to an order of magnitude, achieving a sensitivity of 57 pF per 1% of strain, linearity of 0.99, and drift below 1.5% after 500 cycles in the model sensors. On the other hand, these results suggest that at least certain combinations of monomers have adverse effects on the functional properties due to the displacing effect. Finally, the dielectric properties were linked to a simple strain-sensing test based on capacitance measurements, revealing nontrivial coupling between the dielectric parameters and the sensor performance.

## Supplementary Material



## Notes

Dielectric data as a function of frequency for all samples and Jacobs working curves for all samples (PDF)
