# Supplementary material for: Copolymer Considerations on the Displacing Effect for the Effective Functionalization of 3D-Printed Photopolymer Sensors with Carbon Nanotubes
Source: ACS Omega. 2026 Apr 2;11(14):21805–15. doi: 10.1021/acsomega.5c11529 (PMC13084506; doi:10.1021/acsomega.5c11529)
Supplement: Supplementary file 1 [file ao5c11529_si_001.pdf]

Copolymer considerations on the displacing effect for effective functionalization of 3D-printed photopolymer sensors with carbon nanotubes

Veronika Sevriugina[a], Petr Lepcio[a]*

[a]Central European Institute of Technology, Brno University of Technology, Purkyňova 656/123, 612 00 Brno, Czech Republic

*petr.lepcio@ceitec.vutbr.cz

Supplementary Information

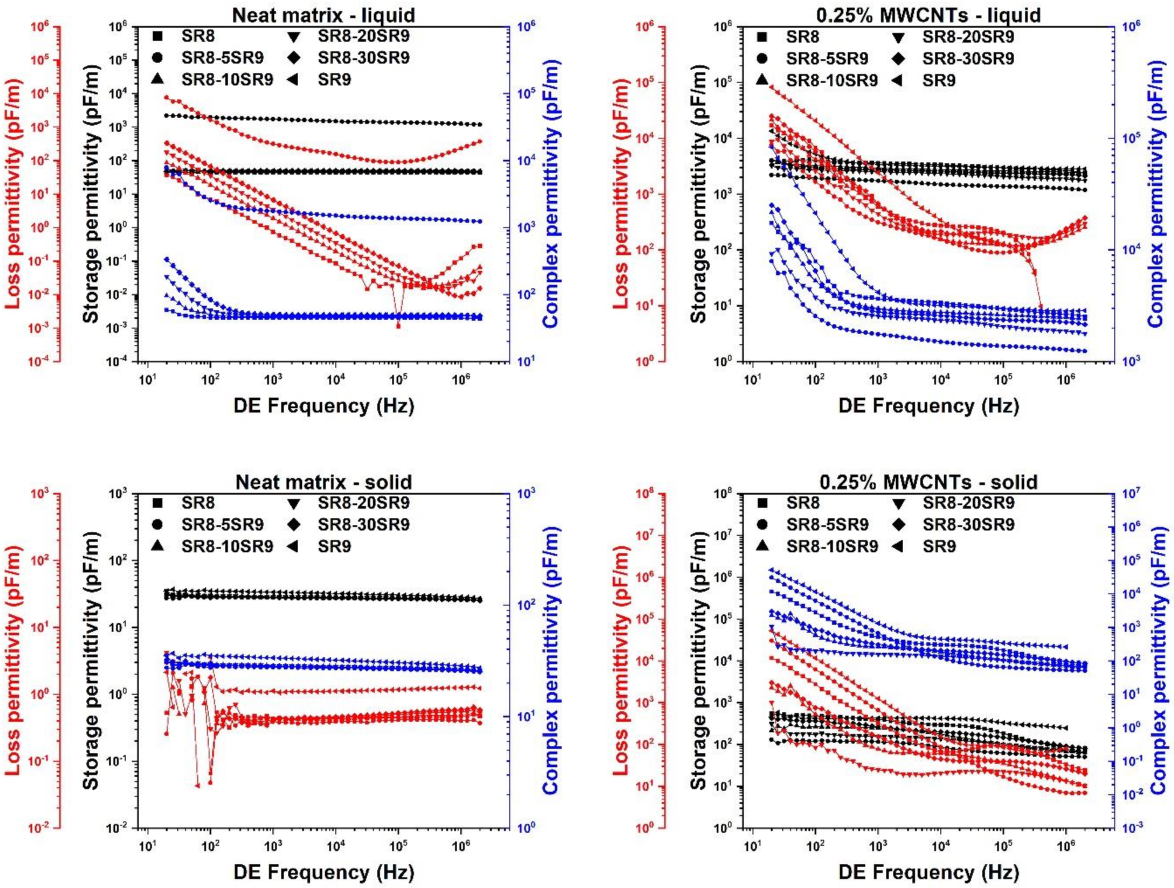

Figure S1: Dielectric data as a function of frequency.

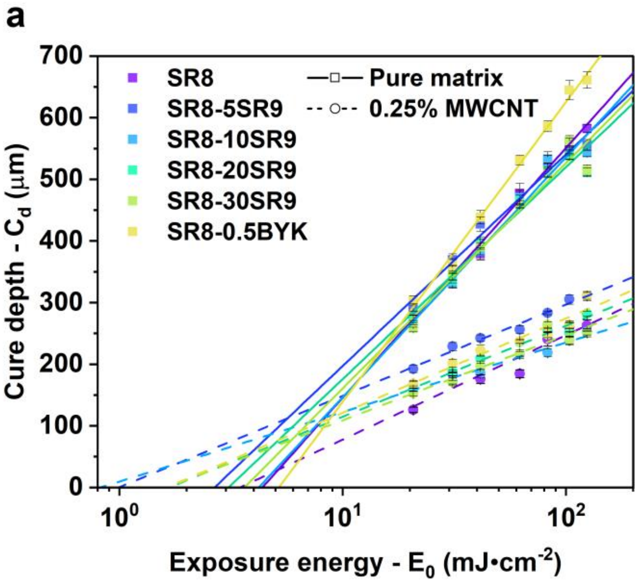

Figure S2: Jacobs working curves
